# Comparison of Pressure Control Ventilation and Palpation Methods to Protect the Endotracheal Tube Cuff During Tracheotomy: A Manikin Simulation Study

**DOI:** 10.7759/cureus.53214

**Published:** 2024-01-30

**Authors:** Yuki Kojima, Takeshi Murouchi, Kazuma Asano, Kazuya Hirabayashi

**Affiliations:** 1 Anesthesiology, Asahi General Hospital, Asahi, JPN; 2 Dental Anesthesiology, Asahi General Hospital, Asahi, JPN; 3 Dentistry and Oral Surgery, Asahi General Hospital, Chiba, JPN

**Keywords:** endotracheal tube, general anesthesia, tracheotomy, pressure control ventilation method, palpation method

## Abstract

Objective

This study aims to evaluate and compare the suitability and safety of palpation and pressure control ventilation (PCV) methods for the accurate positioning of an endotracheal tube (ETT) cuff.

Methods

We conducted a pilot simulation randomized crossover study using a medical manikin. Twenty junior resident physicians who had completed anesthesiology training participated in the study. Airway management was performed using a modified manikin designed to simulate palpation and PCV methods. Participants performed both methods in a randomized order. The primary outcome was the number of successful ETT placements. The secondary outcomes were procedure duration and the perceived difficulty of each procedure.

Results

Five successful procedures were observed in the palpation method group (PALG), while 19 were observed in the PCV method group (PCVG). The duration of the trial was 98 s (standard deviation [SD], 41) in the PALG and 93 s (SD, 49) in the PCVG. The visual analog scale score for difficulty encountered during the trial was 30 (SD, 21) in the PALG and 69 (SD, 19) in the PCVG.

Conclusions

Our findings suggest that the PCV method had a higher success rate than the palpation method. Thus, the PCV method may be more suitable for inexperienced physicians to perform the procedure with greater confidence.

## Introduction

Tracheostomy can be performed under local or general anesthesia [1-3]. In recent years, tracheostomy have been increasingly performed under general anesthesia due to the risk of aerosol infection [4,5]. However, care should be taken to avoid damage to the endotracheal tube (ETT) cuff when performing the tracheostomy under general anesthesia. Physicians must carefully set the depth of the cuff, as placement of the ETT cuff too deep induces unilateral ventilation, whereas extensively shallow placement increases the risk of damage and extubation. Among patients with poor respiratory function, unilateral ventilation may lead to difficulty maintaining oxygenation and ventilation. Fiberscopes have also been used to confirm the position of the ETT cuff but carry a risk of aerosol contamination [6]. Considering that the entire operating room is contaminated by aerosols, we believe that using a fiberscope should be avoided as much as possible.

Aside from a fiberscope, two methods can be used to ensure accurate positioning of the ETT cuff during ventilation: the palpation and pressure control ventilation (PCV) methods [7-9]. In the palpation method, the position of the tube is confirmed by pressing the cuff percutaneously, whereas in the PCV method, the position of the tube is set using a respirator. The palpation method necessitates the compression of the cuff from above the trachea, presenting a risk of potential damage to the trachea and the occurrence of bucking. The PCV method, where the cuff remains inflated until single-lung ventilation is achieved as the trachea is advanced, also poses a risk of damage to the trachea. Each technique carries its own risk of complications, and to date, no studies have been reported comparing them in terms of higher success rates and safety.

We hypothesized that the PCV method would ensure higher success rates for tracheostomy than the palpation method. We conducted a pilot simulation randomized crossover study using a manikin to evaluate and compare the suitability and safety of both methods.

## Materials and methods

Study design

This randomized crossover manikin study adhered to the applicable Consolidated Standards of Reporting Trials (CONSORT) guidelines and was approved by the ethics committee of Asahi General Hospital (approval number: 011810). This study was prospectively registered on a publicly accessible database (UMIN Clinical Trials Registry ID: UMIN000047020) and was performed at Asahi General Hospital between March and August 2022. Written informed consent was obtained from all participants.

**Figure 1:**
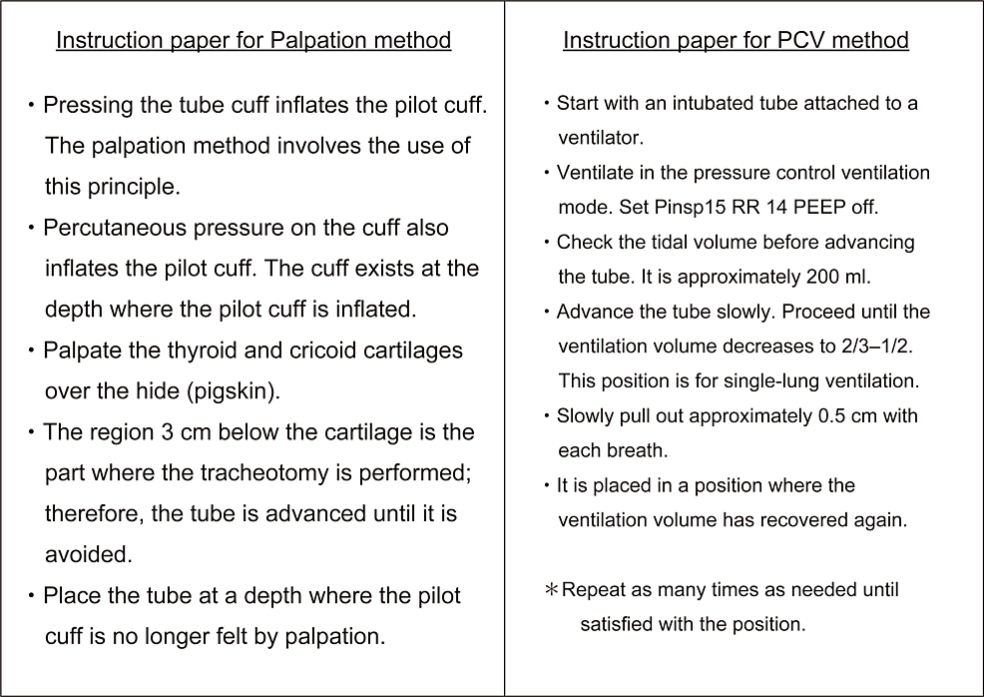
Instructions for each method All participants read the instructions until they completely understood the methods before each trial’s procedure.

This study included 20 junior resident physicians who had completed two months of anesthesiology training. They also underwent the surgical airway access training program. Participants were eligible for inclusion in the trial if they had just completed anesthesiology training within one month. Participants who could not understand the content of the trial even after reading the instructions document were excluded (Figure 1). Before participating in this study, participants were instructed not to engage in lectures or practical exercises related to the setting of tube depth during tracheotomy. It was only through the explanatory materials provided for this study that they first gained an understanding of each method. They were then tasked with applying these methods in practice to manikin models.

**Figure 2:**
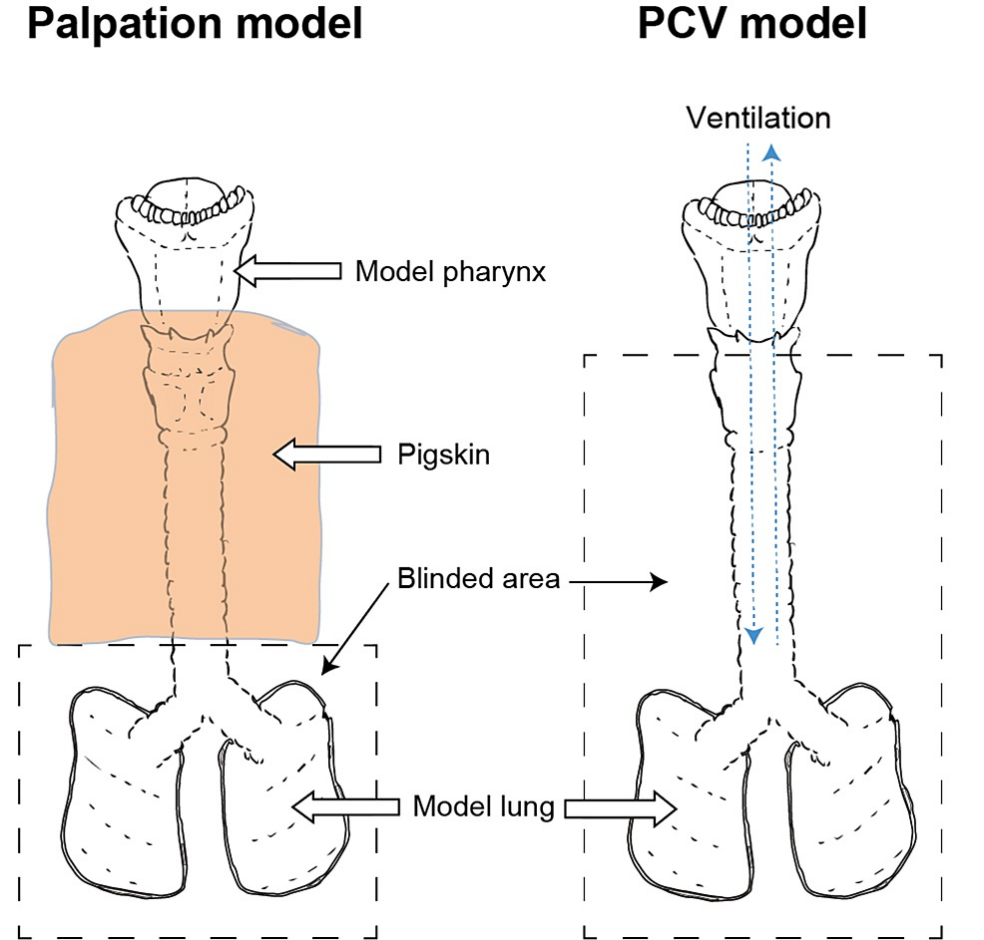
Manikins used as models for the palpation and pressure control ventilation methods Both trials were blinded, such that the tube position was unknown.

All participants read the instructions and watched a demonstration video for the standardization of methods before performing the measurements. First, all participants were to spend five minutes reading the instruction sheet. Second, they watched the instruction video to understand what they would actually perform (Video 1, 2). We used a modified manikin for airway management (Airway Management Trainer, Laerdal, Norway), which we designed to simulate the palpation and PCV methods (Figure 2). Pig skin was applied only in the palpation group, as the PCV group did not require pigskins. Participants in both groups were blinded from visualizing the lung expansion in the manikin. Participants performed both methods in a randomized order, determined using internet-based software (INDICE cloud). There were no restrictions, such as blocking and block size, for randomization. No blinding was performed during the allocation.

**Video 1:** Palpation method Educational video of the practical techniques of the palpation method.

**Video 2:** Pressure control ventilation method Educational video of the practical techniques of the pressure control ventilation method.

**Figure 3:**
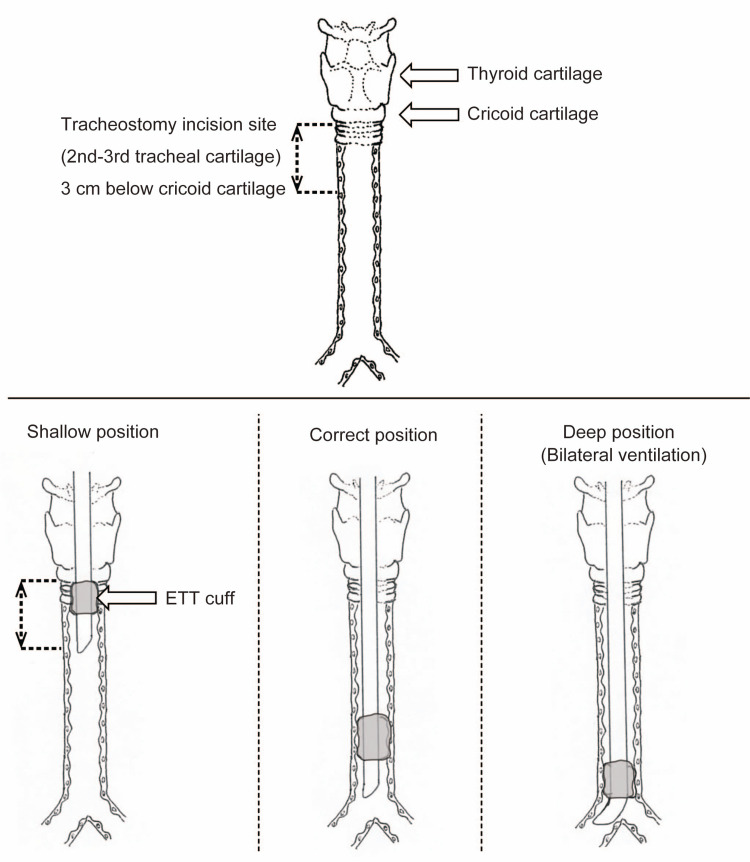
Overview of all the positions of the tracheal tube cuff The ETT cuff should be placed below the position where the incision is made, while avoiding single-lung ventilation.

The primary outcome was the number of successful ETT placements. We assumed that the area of tracheostomy was 3 cm below the cricoid cartilage and therefore evaluated whether the ETT cuff was placed in an appropriate area where it would neither be damaged during the tracheostomy nor induce one-lung ventilation (Figure 3). This length was set because the position of the tracheal cartilage where the tracheotomy is generally performed is approximately 3 cm lower. In actual clinical practice, this distance varies among patients. The secondary outcomes were the duration of the trial and the difficulty level of each method. The trial duration was measured from the time of tracheal intubation to ETT placement.

Participants were asked to determine their position based on their already-intubated state. The tube was set in its shallowest position. The difficulty experienced by the participants in performing both methods was assessed using the visual analog scale (VAS), where 0 indicated “no difficulty” and 100 indicated “maximum difficulty.” The ETT cuff pressure was measured during the trial using SmartCuff (Murata Manufacturing Co., Kyoto, Japan). The trials were performed one week apart. All participants received a questionnaire assessing their perceptions of the methods after the trials.

Data analyses

Two exam monitors evaluated the success or failure of the test after performance. The result of the performance was not communicated to the participants until the clinical study was completed. Statistical analysis was performed using the Wilcoxon signed-rank test (OriginLab Corporation, Northampton, MA, USA) and Fisher’s exact test (R software, The R Foundation for Statistical Computing, Vienna, Austria). Statistical significance was set at P < 0.05, except for carryover effects.

## Results

Data from all 20 participants were included in the study. Table 1 presents the trial results for all participants. Five successful procedures were observed in the palpation method group (PALG), and 19 were observed in the PCV method group (PCVG). Fifteen failed procedures were observed in the PALG, and one was observed in the PCVG. The duration of procedures was 98 s (standard deviation [SD], 41) in the PALG and 93 s (SD, 49) in the PCVG. The maximum cuff pressure was 66 cm H_2_O (SD, 38) and 138 cm H_2_O (SD, 49) in the PALG and PCVG, respectively. The VAS scores for ease encountered during the trial were 30 (SD, 21) in the PALG and 69 (SD, 19) in the PCVG.

**Table 1:** Trial results for all study participants The PCV method resulted in significantly more successful procedures than did the palpation method. The PCV method was also easier to perform than the palpation method.

	PALG (n=20)	PCVG (n=20)	P-value
Successful trials (n)	5	19	<0.01
Fail trials (n)	15	1	<0.01
Duration of the trial (seconds)	93±49	98±41	0.49
Maximum cuff pressure (cm H_2_O)	138±49	66±38	<0.01
The difficulty (VAS)	69±19	30±21	<0.01

Ten participants responded that they lacked confidence while performing the palpation method, as it was subjective and based on tactile sensation. Fifteen participants found the PCV method to be more objective because the position of the ETT could be determined based on numerical values.

## Discussion

The PCV method resulted in significantly more successful procedures and was easier to perform than the palpation method. The palpation method was associated with a lower ETT cuff pressure than the PCV method. We hypothesized that high cuff pressure would likely put a strain on the trachea; therefore, low cuff pressure would place less burden on the trachea. As such, the palpation method may be considered less invasive than the PCV method [10,11]. Further, there was a slight but non-significant difference in procedure duration between the two groups.

The depth of the ETT can also be determined through endoscopy. However, this approach requires disconnection of the circuit during the procedure, which increases the risk of aerosol infection. The palpation and PCV methods are preferable due to their higher level of safety. Furthermore, PCV is the only method that can confirm the tube position until the operation is complete, since palpation cannot be performed once the operation has begun. When physicians are inexperienced in the management of ETT, using methods that are heavily dependent on physician senses and experience may result in low accuracy. In the present study, physicians with respiratory training were able to perform ETT management using the PCV method, which enabled objective assessment. The tracheostomy is a stressful procedure for both surgeons and physicians, as a failed tracheostomy can be life-threatening. Damage to the ETT cuff can cause burns in the respiratory tract due to the use of electric scalpels, poor ventilation during surgery, or aerosol infections [12,13]. The preservation of the tracheal tube's cuff sustains ventilation and minimizes the generation of aerosols that impact the surgeon, thereby preventing the degradation of visibility in the surgical field, which is crucial for the operation's success. This study was executed with participants who had merely received preliminary instructions, indicating that the findings could offer insights into the more efficacious method for physicians inexperienced in adjusting the tube placement during tracheotomy procedures. The ability to safely conclude the surgery has the potential to positively influence patient prognoses.

The results of this Manikin study must be interpreted with caution. In the palpation method model, we used pig skin to simulate human skin. A wide range of situations are encountered in clinical practice, and these may be easier or more difficult to manage than the simulation conditions used in the present study. There are also limitations in the interpretation of the cuff pressure results. As the materials and manikin used in the model are different from those of the human body, whether the measured cuff pressure was similar to the actual clinical values is unknown. It can be posited that maintaining a low cuff pressure is safer; however, this study does not definitively conclude the statistical significance between the PCV and palpation methods in terms of safety. The tracheotomy site was determined as the area up to 3 cm below the cricoid cartilage, yet this parameter could vary among patients due to differences in neck length and the distance from the skin to the trachea. Given the anatomical variations in clinical settings, the efficacy of either method must ultimately be evaluated through clinical research. Prospective interventional studies in cases of tracheotomy under general anesthesia appear to be the most desirable form of clinical research in this context. Although more clinical studies are necessary, it would be difficult to compare the effectiveness of the two procedures clinically. The PCV method, relying less on tactile sensation compared to the palpation method, might be less susceptible to anatomical variations among individuals. This characteristic could potentially render it a more reliable technique in the presence of divergent anatomical structures. As patients have different tracheal lengths and neck morphologies, further simulation research using a more accurate model is warranted.

## Conclusions

We conducted a simulation randomized crossover study with a manikin to compare the safety and suitability of the palpation and PCV methods for ensuring appropriate ETT placement during tracheostomy. Our results indicate that the PCV method is associated with greater accuracy and confidence. However, given that this was a simulation study, there are limitations in the interpretation of its results. Therefore, more clinical and simulation studies are necessary.
